# A comparative study to elucidate factors explaining willingness to use home-care robots in Japan, Ireland, and Finland

**DOI:** 10.1038/s41598-024-79414-y

**Published:** 2024-11-12

**Authors:** Hiroo Ide, Sayuri Suwa, Yumi Akuta, Naonori Kodate, Mayuko Tsujimura, Mina Ishimaru, Atsuko Shimamura, Helli Kitinoja, Sarah Donnelly, Jaakko Hallila, Marika Toivonen, Camilla Bergman-Kärpijoki, Erika Takahashi, Wenwei Yu

**Affiliations:** 1 Institute for Future Initiatives, The University of Tokyo, Hongo 7-3-1, Bunkyo-ku, Tokyo, Japan; 2 Department of Community Health Nursing, Division of Innovative Nursing for Life Course, Graduate School of Nursing, Chiba University, 1-8-1 Inohana, Chuo-ku, Chiba, Japan; 3 Division of Nursing, Faculty of Healthcare, Tokyo Healthcare University, Higashi gotanda 4-1-17, Shinagawa-ku, Tokyo, Japan; 4 UCD School of Social Policy, Social Work and Social Justice, University College Dublin, Hanna Sheehy Skeffington Building, Belfield, D04 N9Y1 Dublin 4, Ireland; 5 Division of Visiting Nursing, School of Nursing, Shiga University of Medical Science, Seta Tsukinowa-cho, Otsu, Shiga Japan; 6 Division of Community Health Nursing, Department of Nursing, Faculty of Health Science, Toho University, Miyama 2-2-1, Funabashi, Japan; 7 Seinäjoki University of Applied Sciences, FI-60101 Seinäjoki, Finland; 8 Humana, Teollisuuskatu, 13 00510 Helsinki, Finland; 9 Graduate School of Humanities, Chiba University, 1-33 Yayoi-cho, Inage-ku, Chiba, Japan; 10 Center for Frontier Medical Engineering, Chiba University, 1-33 Yayoi-cho, Inage-ku, Chiba, Japan

**Keywords:** Home-care robot, Caregivers, Robotics, Research and development, Multi-country study, Health care, Medical research

## Abstract

The implementation of home-care robots is sometimes unsuccessful. This study aimed to explore factors explaining people’s willingness to use home-care robots, particularly among care recipients and caregivers. Surveys were conducted in Japan, Ireland, and Finland. The survey questionnaire comprised four categories (familiarity with robots, important points about home-care robots, functions expected from home-care robots, and ethically acceptable uses), with 48 items assessing users’ willingness to use home-care robots. The responses from 525 Japanese, 163 Irish, and 170 Finnish respondents were analyzed to identify common and distinct factors influencing their willingness to use these robots. Common factors across the countries included “willingness to participate in research and development,” “interest in robot-related news,” and “having a positive impression of robots”. The distinct factors for each country were: “convenience” in Japan; “notifying family members and support personnel when an unexpected change occurs in an older person” in Ireland; and “design” in Finland. Therefore, developers should determine potential users’ willingness to participate in the research and development of home-care robots and consider a system that involves them in the development process.

**Supplementary Information:**

The online version contains supplementary material available at 10.1038/s41598-024-79414-y.

## Introduction

Population ageing is progressing at a rapid pace in developed countries. By 2060, older adults are expected to account for 28.2% of the total populations in Europe, North America, Japan, Australia, and New Zealand^1^. The World Health Organization (WHO) estimates that by 2030, one in six individuals globally will be aged 60 years or older^2^. This suggests that the number of family members and professionals available to provide care for older adults in their homes will be insufficient. The Japanese government reported that an additional 250,000 caregivers will be needed by 2026^3^. Keva, a pension provider for public sector employees in Finland, estimated that there will be shortage of 16,600 nurses, including those caring for older people in 2022^4^. Half of the shortage has occurred in the last two years. Family Carers Ireland found that about two-thirds of family carers felt they could not receive formal support because of inadequate staffing^5^.

Simple, reliable, and effective technologies are needed to mitigate the impact of care workforce shortage and help older adults to age in their homes^6^. One way to overcome this shortage is to develop and deploy “robot technology to help older adults become more independent and reduce the burden on caregivers”^7^. Considering the effective use of the limited workforce, the Japanese Government implemented several programs regarding care robots. In November 2012, the Ministry of Health, Labour and Welfare and the Ministry of Economy, Trade and Industry formulated the “Priority Areas for the Use of Robotics in Long-Term Care,” which included home-care; it was revised in 2014^8^. However, the development and sale of several highly anticipated care robots was discontinued because of difficulty in developing products that could meet the needs of Japanese long-term care settings^9^. This is not unique to Japan. Care robots were developed and widely touted in the media, but failed to sell in Finland too^10^. Gaining social acceptance for such assistive home-care robots remains a common issue in ageing societies^11–14^. Even with the support and recommendations of government agencies and technology developers, the use of care robots on an appropriate scale would be difficult to attain and sustain^15^.

Studies have shown that in spite of technological advancement in assistive technologies, the acceptance of robots among older adults remains low^16–19^. Recently, Maibaum et al.^20^, in their critique of care robots in healthcare, argued that “there has been a fundamental conflict between economic, professional, and ethical care concepts and that this care conflict shapes the phenomenon of care robots” (p. 470). The circumstances in which home-care robots are used vary according to users’ mental and physical conditions, home environment, and financial situation. These factors are related to the inner setting domain of the Consolidated Framework for Implementation Research (CFIR), which assesses multiple factors influencing the implementation of innovations^21,22^. The current CFIR defines “recipient-centeredness,” focusing on the needs of the recipient (patient), as a concept similar to the “user-centric principle.” Given that the user of CFIR is the developer, what more specific considerations should they take into account?

Several components of user-centric principles have been addressed in previous studies on technology acceptance models^23,24^. Theoretical models explain the public’s technology acceptance. Some models, such as the Almere model (AM), determine user acceptance of social assistive robots for older adults^23^. To the best of our knowledge, Alaiad and Zhou^24^ were the first to introduce ethical concerns in the adoption of home healthcare robots, using a technology acceptance model (a structural model of determinants; SMD). They raised issues of privacy—referring to the lack of control over the collection and use of personal information—and ethical concerns—defined as a standard of behavior and a concept of right and wrong beyond legal concerns. A previous study elucidated the relationship between the willingness to use home-care robots and ethical issues, including personal information and privacy protection, which were similar to Alaiad and Zhou’s concept of privacy concerns^25^. Another component of the user-centric principle is the participation of the user in research and development^26^. Indeed, users’ willingness to participate in research and development can potentially encourage willingness to use home-care robots^25^. A systematic review showed that NIHR public involvement and engagement project (INVOLVE) in the UK facilitated public involvement in health care research, which had a positive impact on the enrolment of study participants^27^. INVOLVE had broad impacts such as improving research quality and design, fostering trust and empowerment among the public, and increasing awareness of the ethical and moral imperatives of researchers.

This study examined how much user-centric principles affect users’ willingness to use home-care robots, compared to other factors such as users’ attributes, past experience, and features and functions of the robots. Thus, first, this study aimed to explain the general structure of users’ willingness to use home-care robots. Second, it aimed to address whether the user-centric principles are fundamental and universal across countries. Therefore, we conducted surveys in Japan, Ireland, and Finland. Despite differences in population ageing rates among the three countries, we found common challenges related to population ageing; furthermore, all three countries have national strategies for ageing (Table 1).

**Table 1:** Demography and national strategies in for ageing Japan, Ireland, and Finland.

	Japan	Ireland	Finland
Population (thousand) ^a), b)^	125,502	5,011	5,541
Percentage of people aged 65 and over ^a), b)^	28.9%	19.1%	22.9%
Historical outlines of national strategies	Long-term Care Insurance scheme (2000) Community-based integrated care system “The Orange Plan” (5-year dementia measures) (2012) “The New Orange Plan” (2015) Guidelines regarding decision-making support for people with dementia (2018) The Dementia Policy Promotion Charter (2019) The Dementia Basic Law (2024)	Historically, institutional care has been national standard. The Home Care Package Scheme (2006) The Nursing Home Support Scheme (2009) The National Positive Ageing Strategy (2013) The Dementia Strategy (2014) The Assisted Decision-Making (Capacity) Act (2015) The National Health Reform Programme (Sláintecare) (2018)	The Socially Sustainable Finland 2020: Strategy for Social and Health Policy was published (2011) The National Memory Program 2012–2020 (national dementia strategy) (2012) The National Key Project for Home-Care and Informal Care (2016–2019) National Programme on Ageing 2030: For an age-competent Finland (2020)

^a)^ All figures of the three countries are for the year 2021. Data from OECD (2023), Population (indicator), 10.1787/d434f82b-en (accessed 25 April 2024).

Ireland’s demographic information derived from the Census of Population 2016. https://www.cso.ie/en/releasesandpublications/ep/p-cp3oy/cp3/agr/.

## Results

### Demography of the respondents

A total of 1,004 potential users of home-care robots responded to the questionnaire: 528 from Japan, 296 from Ireland, and 180 from Finland. As willingness to use a home-care robot was the dependent variable for the analysis, responses that left the question item unanswered were excluded from the analysis. Consequently, data from 525 Japanese, 163 Irish, and 170 Finnish respondents were analyzed (Table 2).

**Table 2:** Descriptive statistics of the survey participants.

		Japan *n* = 525		Ireland *n* = 163		Finland *n* = 170		
		n	(%)	n	(%)	n	(%)	*p*-value ^a)^
Attribute	Older adults ^b)^	175	(33.3)	101	(62.0)	98	(57.6)	< 0.001
	Family caregivers ^b)^	167	(31.8)	37	(22.7)	81	(47.6)	< 0.001
	Home-care professionals ^b)^	317	(60.4)	54	(33.1)	66	(38.8)	< 0.001
Sex	Male	121	(23.0)	33	(20.2)	51	(30.0)	< 0.001
	Female	403	(76.8)	114	(69.9)	115	(67.6)	
	Other or unanswered	1	(0.2)	16	(9.8)	4	(2.4)	
Age (years)	Under 39	57	(10.9)	15	(9.2)	26	(15.3)	< 0.001
	40-44	50	(9.5)	13	(8.0)	9	(5.3)	
	45-49	53	(10.1)	8	(4.9)	10	(5.9)	
	50-54	66	(12.6)	5	(3.1)	6	(3.5)	
	55-59	67	(12.8)	9	(5.5)	10	(5.9)	
	60-64	57	(10.9)	8	(4.9)	8	(4.7)	
	65-69	43	(8.2)	13	(8.0)	23	(13.5)	
	70-74	37	(7.0)	24	(14.7)	23	(13.5)	
	75-79	40	(7.6)	21	(12.9)	16	(9.4)	
	80-84	28	(5.3)	18	(11.0)	14	(8.2)	
	85 and over	27	(5.1)	17	(10.4)	20	(11.8)	
	unanswered	0	(0.0)	12	(7.4)	5	(2.9)	

^a)^ *p*-values calculated by χ² test.

^b)^ Since participants comprised not only people with a single attribute but also those with multiple attributes, such as being an older adult and family caregiver, the sums of the numbers of each attribute were not equal to the total number of participants in each country. The denominators of the percentages of each attribute were 525 for Japan, 163 for Ireland, and 170 for Finland.

Missing values were imputed by question items 2–14 for Japanese respondents, 2–25 for Irish respondents, and 1–16 for Finnish respondents Therefore, the number of respondents for whom data were imputed per question item ranged from 0.4 to 2.7% of the Japanese, 1.2–15.3% of the Irish, and 0.6–9.4% of the Finnish participants. By country, the largest difference in mean score for every item after imputation was ± 0.010 for Japan, ± 0.050 for Ireland, and ± 0.030 for Finland. The differences for the standard deviations were smaller: -0.010 for Japan, -0.063 for Ireland, and − 0.043 for Finland (Appendix Table 1).

### Analysis

As there was no high correlation among the items based on correlation analyses, we included all items in the bivariate analysis. In the bivariate analysis for all three countries, many items—including all four items for ethically acceptable uses—were significantly associated with the willingness to use a home-care robot (Appendix Table 2). Significant items in all three countries were “I am interested in robot-related news” and “I have a positive impression of robots” for familiarity with robots, and “willingness to participate in research and development” for ethically acceptable uses (Appendix Table 3).

**Table 3:** Factors affecting the willingness to use home-care robots: multivariate regression analysis.

Independent variable			Japan *n* = 525			Ireland *n* = 163			Finland *n* = 170	
		β	SE	VIF	β	SE	VIF	β	SE	VIF
**Familiarity with robots**										
	I am interested in robot-related news.	0.262	0.053	1.364	0.445	0.066	1.272	0.212	0.066	1.385
	I have a positive impression of robots.	0.217	0.048	1.090	0.205	0.064	1.166	0.319	0.059	1.250
**Important points about home-care robots**										
	Convenience	0.163	0.054	1.184						
	Design							0.246	0.064	1.073
**Functions expected from home-care robots**										
	Notifying family members and support personnel when an unexpected change occurs in an older person				0.150	0.080	1.334			
	Providing support for the movement/mobility of older people in their daily lives	0.132	0.049	1.188						
**Ethically acceptable uses**										
	Willingness to participate in research and development	0.188	0.033	1.312	0.222	0.055	1.432	0.263	0.053	1.420
	Adjusted R^2^		0.381			0.531			0.498	

β: Standardized coefficients; SE: Standard error; VIF: Variance inflation factors.

Multivariate regression analysis identified the items that predicted the willingness to use a robot (Table 3). Adjusted R^2^ were 0.381 to 0.531 and none of the relationships between the items had a VIF of 4 or greater. For ethically acceptable uses, the “willingness to participate in research and development” item was a significant factor in all three countries(Japan: 0.188[standard error (SE) = 0.033], Ireland: 0.222[SE = 0.055], Finland: 0.263 [SE = 0.053]), as were “I am interested in robot-related news” (Japan: 0.262[SE = 0.053], Ireland: 0.445[SE = 0.066], Finland: 0.212 [SE = 0.066]) and “I have a positive impression of robots” (Japan: 0.217[SE = 0.048], Ireland: 0.205[SE = 0.064], Finland: 0.319 [SE = 0.059]) for familiarity with robots.

Distinct items were the following. Japanese respondents’ willingness to use home-care robots were “convenience” for important points about robots; and “providing support for movement/mobility of older people in their daily lives” for functions expected from home-care robots. Irish individuals’ willingness to use these robots was influenced by the expectation that they would “notify family members and support personnel when an unexpected change occurs in an older person” for functions expected from home-care robots. The Finnish participants emphasized the importance of “design” when considering home-care robots (Table 3).

## Discussion

This study explored the willingness to use home-care robots among potential users in Japan, Ireland and Finland. We found commonalities in the use of home-care robots that empirically support the concept of user-centric principles.

We sequentially discuss the implications of the results derived for the three countries. The final analysis for predicting the willingness to use a home-care robot in Japan included two items related to familiarity with robots: interest in robot-related news and having a positive impression of robots. Thus, in Japan, subjective aspects were identified as unique factors.

Home-care robot development in Japan is being encouraged by the government, industry, and academia, and robot-related marketing is being used to increase the demand for robots, according to robotic anthropology research^28,29^. In other words, rather than the Japanese being culturally pre-disposed to feeling amicable towards robots, the society strives to align its culture to become robot-friendly^30^. Compared to Western Europe, East Asia has always been viewed as being more inclined towards “techno-optimism”^31^. Consequently, government officials and the developers of home-care robots have the techno-optimistic belief that robots are the solution to home-care in an ageing society. Hence, in Japan, robot-related news may be designed to increase the demand for these robots because people with an interest in robot-related news are more susceptible to influence on this subject.

Lolich et al.^32^ state that Ireland is presently behind in its uptake of robotics in general. However, vigorous investment is expected to accelerate the uptake to bridge the digital divide. In 2021, the population ageing rate in Ireland was 14.8%^33^; it has increased by 35% since 2013, considerably higher than the EU average increase of 17.3%^34^. Given this unique situation, the finding that interest in robot-related news relates to willingness to use a home-care robot suggests a strong inclination of respondents towards home-care robots.

Several stakeholders in Ireland perceive healthcare policies, such as Sláintecare, as drivers embedding artificial intelligence and robotics into health and social care in the country^35^. In May 2021, the government published the Sláintecare Implementation Strategy and Action Plan 2021–23, which emphasized the role of technology and digital innovations to integrate care pathways. If successfully implemented, safe and timely access to care will be enabled for older people. In the past few years, there has been a significant emphasis on humanoid robots, such as Stevie and Mylo, that have been featured in domestic and foreign media. Thus, the expectations may reflect an overblown image of robots as popular, which contrasts with their low production and usage rates.

In Finland, “design” was associated with the willingness to use a home-care robot in the statistical analysis. Finnish design is well known globally and is important in robot manufacturing^36^. Additionally, the “capacity to increase mental and physical wellbeing and comfort” was a significant factor in logistic regression analysis. Finland has advanced information and communication technology (ICT) manufacturing, which uses robots for logistics^37^. Accordingly, a proposal entitled Robotics in Care Services: A Finnish Roadmap^38^—which cites the conceptual framework for dignity from a study of nursing home residents by Pleschberger^39^ —argues for the importance of interpersonal dignity when developing robots for use in care services. As for what should be considered when adopting a robot, Finnish respondents’ selection of “capacity to increase mental and physical wellbeing and comfort” reveals that they hold strong user-centered values, possibly because of this proposal.

Both the “ethically acceptable uses” and “familiarity with robots” were common factors across the three countries. For the first time, robots are being used to help in the health of humans^40^. Japan, Ireland, and Finland have different historical, cultural, demographic, and economic backgrounds. Our results demonstrated both commonality and unique aspects for each country. Unsurprisingly, familiarity with robots is an important factor. Although it is difficult to explain why user participation in the research and development process is a common factor, it is likely crucial for improving societal acceptance of home-care robots.

Therefore, a system that encourages participation in research and development of home-care robots is needed. Lolich et al.^32^ argue that the successful adoption of home-care technology requires an understanding of the user’s social position, from the economic, political, emotional, and cultural value perspectives. Pilotto et al.^12^ also suggest the significance of interdisciplinary collaboration by researchers, developers, and end-users to implement technologies aimed at meeting older people’s needs. To this end, conducting empirical studies would be more informative for the future. One study reported participants’ changes in understanding the robot through a year-long, seven stage co-design process with 28 older people^41^. A Finish study focused on conducting participatory research by using action research and living lab methodologies to develop and apply robots in care for older people^42^. Developers across countries should be aware of this significance and consider developing a system that involves potential users in the development process.

This study has some limitations. First, data were collected between November 2018 and February 2019. Since then, both people and society’s perceptions of robots and other technologies may have changed because of their experience with the COVID-19 pandemic. The environment surrounding home-care robots is likely to change due to emerging infectious diseases, aging populations, and developing technologies such as ICT and artificial intelligence. These changes must also be considered when conducting user-centric research and development. Second, other countries involved in the development and manufacture of robots, such as the U.S., China, and Germany, were not included in the study. Third, the number of survey participants in the three countries was not sufficient to analyze each demographic (older adults, family caregivers, or home-care professionals). Even if commonalities in the willingness to use home-care robots were found across countries, differences may exist because of these attributes. Finally, this study has a self-report bias because it describes the home-care robot briefly and presents images. Recent studies aimed at explaining the acceptability of care robots have used vignettes describing detailed use settings of the care robots^43,44^. Developing more-reliable methods for clarifying users’ perceptions of home-care robots remains a challenge.

Our findings indicate that potential users’ intention to participate in research and development processes was associated with their willingness to use home-care robots. However, not many people can actually participate in the research and development process. Therefore, it is necessary for developers to be more proactive in keeping user-centric principle in mind. In the future, the biggest challenge may be to create a system that involves older adults, family caregivers, and care professionals and develop home-care robots that users truly need in their daily lives, while considering the potential risks and benefits.

## Methods

### Survey

**Fig. 1:**
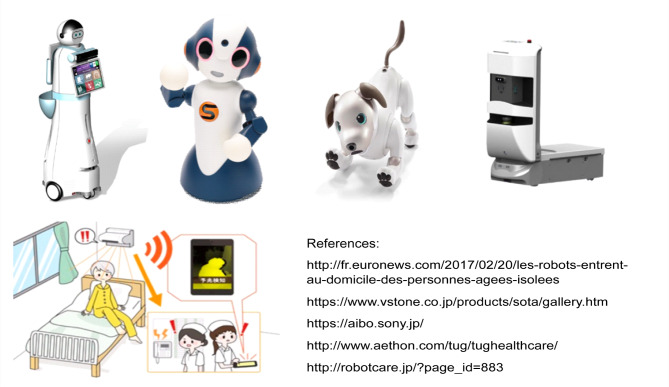
Images of home-care robots provided in the questionnaire. The figure included typical images of robots. We presented the figure to the study subjects with the following definition of home-care robots in the questionnaire: “Home-care robots come in many forms. The term “home-care robot” used in this survey is a general expression for devices and systems that perform functions such as monitoring older people and their surroundings and providing support for older people and/or their caregivers (including communication that enables interactive conversation, assistance with activities of daily living, or managing medications).”

We developed a conceptual framework of factors that could potentially determine the willingness of users to use a home-care robot. In this framework, willingness to use a home-care robot (willingness to use one to care for a family member, for personal care, and for older adults) were determined by sex; age; experience with robots; ethical principles of respect for patient autonomy, non-maleficence, and beneficence; and features and functions of home-care robots to meet the needs of the older people. Details regarding the preparation of the survey questionnaire are described in Suwa et al.^45^ and Tsujimura et al.^46^. The survey questionnaire comprised four categories—familiarity with robots, important points about home-care robots, functions expected from home-care robots, and ethically acceptable uses, with a total of 48 items (Appendix Table 1). The questionnaire included typical images of robots alongside the definition (Fig. 1).

We conducted surveys in Japan, Ireland and Finland between November 2018 and February 2019. Details regarding the survey procedure are described in Suwa et al.^47^ and Kodate et al.^48^. A brief description of the survey procedure in each country is provided below.

#### Japan

The survey respondents were people aged 65 years and over, family caregivers, and home-care professionals in a prefecture. In 2018, the ageing rate in this prefecture was 28.1%, similar to the national average. We systematically sampled home-care offices from those listed in the government’s Long-Term Care Insurance service information disclosure system. We distributed the questionnaire to 1,936 older people, 2,652 family caregivers, and 1,073 home-care professionals. Those aged 65 years and over as well as family caregivers received the questionnaire forms from the home-care professionals. An online version of the questionnaire was also prepared, so the respondents could answer either by post or via the Internet.

#### Ireland

The responses were collated from three groups. Age Action Ireland distributed the questionnaire to their members (*N* = 1,154), who included people aged 65 years and over who were or might possibly be using health or social care services, and family caregivers who were or might possibly be using services related to nursing care. They completed the questionnaire and sent it back to the researchers. The Irish Gerontological Society (IGS) agreed to distribute the questionnaires to home-care/health and social care professionals, including nurses and care providers. An email including a link to an online version of the questionnaire was sent to the respondents, who answered the questionnaire via the Internet.

#### Finland

The respondents were potential users of home-care robots aged 65 years and over, family caregivers, and home-care professionals in two regions. The questionnaires were distributed by mail in cooperation with the municipal governments. We distributed 1,405 questionnaires. People aged 65 years and over and family caregivers completed the questionnaire and sent it back to the researchers, whereas the home-care professionals could answer the questionnaire either by post or via the Internet.

### Data preparation

The survey items presented to the participants are shown in Appendix Table 1. Respondents were asked to rate their willingness to use a home-care robot if they were to begin receiving long-term care at home. We employed a 4-point Likert scale, rather than a 5-point one, to avoid “either” or “neither” responses, and to conduct further bivariate analysis (1: I would like to use one, 2: I would rather use one than not, 3: I would rather not use one, or 4: I would not want to use one). For the bivariate analysis, a binary variable “willingness to use a home-care robot” was created by converting ratings 1 and 2 into “1: yes” and ratings 3 and 4 into “0: no.”

For the independent variables, responses comprised four categories: familiarity with robots, important points about home-care robots, functions expected from home-care robots, and ethically acceptable uses. The questionnaire comprised 38 items in the following categories: familiarity with robots (How familiar are you with robots?; 7 items rated from 1: not at all to 4: very familiar), important points about home-care robots (To what extent do you place importance on the items below in regard to home-care robots?; 16 items rated from 1: not important to 4: important), and functions expected from home-care robots (To what extent do you think it would be useful for home-care robots to provide the types of support indicated below?; 15 items rated from 1: not expected to 4: expected). For the bivariate analysis, we converted the variables into binary variables by combining ratings 1 and 2 and ratings 3 and 4.

Confirmatory factor analysis based on a previous study confirmed that “ethically acceptable uses” had a 4-factor structure from 10 items (Rated from 1: unacceptable to 4: acceptable)^25^. The Cronbach’s alpha coefficients for the four factors—acquisition of personal information, use of personal information for medical and long-term care, secondary use of personal information, and willingness to participate in research and development—were 0.755, 0.832, 0.887, and 0.844, respectively. We calculated the mean and quantile values for each of the four factors (collection of personal information, usage of personal information for medical or nursing care, usage of secondary personal information, and willingness to participate in research and development). These values were used as a threshold when we converted the variables into binary and quantile variables.

### Analysis

We computed the descriptive statistics of the survey participants from the three countries. For the dependent and independent variables, we imputed missing values using the linear regression approach. Descriptive statistics were computed for each factor before and after imputation to verify that the means and standard deviations after imputation remained in close approximation. The descriptive statistics of the dependent and independent variables were compared across the three countries using the χ² test. Cronbach’s alpha coefficients were calculated for each category by each country.

Factors associated with willingness to use a home-care robot for each country were identified in the following three stages. First, associations between willingness to use a home-care robot and the items were identified using the χ² test and Fisher’s exact test for categorical variables, and t-test for continuous variables. Second, to identify significant items for each category, a logistic regression analysis with binary independent and binary dependent variables was performed after checking the correlations among the items. Finally, we conducted a multivariate regression analysis using a 4-point scale or quantile independent variables and a 4-point scale dependent variable. We tested for adverse effects from multicollinearity between the variables using the criterion of a variance inflation factor (VIF) of 4 or greater^49^; goodness of fit was tested using adjusted R^2^. The analyses were performed using SPSS version 28. The statistical significance level was set at 5%.

### Ethical considerations

All study procedures were conducted in accordance with the guidelines and regulations of each institution and country. The study was reviewed and approved by the ethics review committees in each country: Chiba University Graduate School of Nursing Ethics Review Committee (No. 30-19) in Japan, University College Dublin’s Human Research Ethics Committee - Humanities (HS-18-81- KODATE) in Ireland, and the City of Seinäjoki and the Joint Municipal Authority of Ilmajoki and Kurikka (JIK), both in Finland. We provided a written explanation of the study in accordance with the guidelines and regulations. Informed consent was obtained from all respondents and/or their legal guardians before answering the questionnaire.

## Electronic supplementary material

Below is the link to the electronic supplementary material.

Supplementary Material 1

## Data Availability

Data are available from the authors upon reasonable request. Please contact the corresponding author (suwa-sayuri@faculty.chiba-u.jp).
