# Supplementary material for: A comparative study to elucidate factors explaining willingness to use home-care robots in Japan, Ireland, and Finland
Source: Sci Rep. 2024 Nov 12;14:27656. doi: 10.1038/s41598-024-79414-y (PMC11557874; doi:10.1038/s41598-024-79414-y)
Supplement: Supplementary file 1 — Supplementary Material 1 [file 41598_2024_79414_MOESM1_ESM.docx]

**A comparative study to elucidate factors explaining willingness to use home-care robots in Japan, Ireland, and Finland**

Hiroo Ide, Ph. D, Sayuri Suwa, Ph. D, Yumi Akuta, Ph. D, Naonori Kodate, Ph. D, Mayuko Tsujimura, Ph. D, Mina Ishimaru, Ph. D, Atsuko Shimamura, Ph. D, Helli Kitinoja, MNSc, Sarah Donnelly, Ph. D, Jaakko Hallila, Ph. D, Marika Toivonen, MHS, Camilla Bergman-Kärpijoki, MHC, Erika Takahashi, Ph. D, and Wenwei Yu, Ph. D.

**Appendix Table 1. Descriptive statistics of questionnaire items before and after the imputation of missing values**

|  |  |  | **Before imputation** | | | **After imputation** | | |
| --- | --- | --- | --- | --- | --- | --- | --- | --- |
|  |  |  | **n** | **mean** | **SD** | **n** | **mean** | **SD** |
| **Familiarity with robots** | | | | | | | | |
|  | I have seen news about robots. | Japan | 523 | 3.18 | 0.63 | 525 | 3.18 | 0.62 |
|  |  | Ireland | 161 | 3.07 | 1.00 | 163 | 3.07 | 0.99 |
|  |  | Finland | 169 | 3.05 | 0.98 | 170 | 3.05 | 0.98 |
|  | I am interested in robot-related news. | Japan | 522 | 3.15 | 0.73 | 525 | 3.15 | 0.73 |
|  |  | Ireland | 157 | 3.13 | 0.88 | 163 | 3.12 | 0.87 |
|  |  | Finland | 167 | 2.67 | 0.97 | 170 | 2.68 | 0.96 |
|  | I have seen a robot that was developed for caregiving. | Japan | 518 | 1.97 | 1.04 | 525 | 1.97 | 1.03 |
|  |  | Ireland | 156 | 2.08 | 1.17 | 163 | 2.07 | 1.15 |
|  |  | Finland | 166 | 2.04 | 1.13 | 170 | 2.04 | 1.11 |
|  | I have used a robot that was developed for caregiving. | Japan | 514 | 1.33 | 0.70 | 525 | 1.33 | 0.70 |
|  |  | Ireland | 152 | 1.22 | 0.65 | 163 | 1.20 | 0.63 |
|  |  | Finland | 167 | 1.24 | 0.59 | 170 | 1.24 | 0.59 |
|  | I have seen an anime/manga in which a robot appeared. | Japan | 517 | 2.62 | 1.09 | 525 | 2.62 | 1.08 |
|  |  | Ireland | 153 | 2.27 | 1.35 | 163 | 2.26 | 1.31 |
|  |  | Finland | 168 | 2.36 | 1.28 | 170 | 2.35 | 1.27 |
|  | I have a positive impression of robots. | Japan | 514 | 3.19 | 0.72 | 525 | 3.18 | 0.71 |
|  |  | Ireland | 150 | 2.85 | 0.89 | 163 | 2.87 | 0.85 |
|  |  | Finland | 166 | 2.60 | 1.03 | 170 | 2.61 | 1.02 |
|  | I have used a robot such as an animal-like robot, humanoid robot, or cleaning robot. | Japan | 519 | 1.69 | 1.02 | 525 | 1.70 | 1.01 |
|  |  | Ireland | 153 | 1.28 | 0.81 | 163 | 1.26 | 0.78 |
|  |  | Finland | 169 | 1.44 | 0.91 | 170 | 1.44 | 0.90 |
|  |  |  |  |  |  |  |  |  |
| **Important points about home-care robots** | | | | | | | | |
|  | Convenience (ease of use) | Japan | 517 | 3.69 | 0.66 | 525 | 3.70 | 0.66 |
|  |  | Ireland | 152 | 3.28 | 0.92 | 163 | 3.26 | 0.89 |
|  |  | Finland | 163 | 3.50 | 0.82 | 170 | 3.49 | 0.81 |
|  | Entertainment value (fun to use) | Japan | 518 | 3.06 | 0.89 | 525 | 3.06 | 0.88 |
|  |  | Ireland | 145 | 2.74 | 0.83 | 163 | 2.77 | 0.79 |
|  |  | Finland | 161 | 2.71 | 0.85 | 170 | 2.73 | 0.83 |
|  | Economic efficiency (low running cost) | Japan | 520 | 3.77 | 0.54 | 525 | 3.77 | 0.53 |
|  |  | Ireland | 152 | 3.13 | 0.89 | 163 | 3.12 | 0.86 |
|  |  | Finland | 162 | 3.33 | 0.82 | 170 | 3.32 | 0.80 |
|  | Safety (warranty, after-service) | Japan | 519 | 3.85 | 0.47 | 525 | 3.86 | 0.47 |
|  |  | Ireland | 148 | 3.34 | 0.89 | 163 | 3.31 | 0.86 |
|  |  | Finland | 161 | 3.55 | 0.81 | 170 | 3.54 | 0.79 |
|  | High performance and capability | Japan | 520 | 3.77 | 0.53 | 525 | 3.77 | 0.53 |
|  |  | Ireland | 144 | 3.31 | 0.92 | 163 | 3.28 | 0.87 |
|  |  | Finland | 161 | 3.18 | 0.92 | 170 | 3.17 | 0.90 |
|  | Confidentiality (privacy protection) | Japan | 520 | 3.61 | 0.68 | 525 | 3.61 | 0.68 |
|  |  | Ireland | 148 | 3.29 | 0.94 | 163 | 3.26 | 0.90 |
|  |  | Finland | 163 | 3.50 | 0.84 | 170 | 3.48 | 0.83 |
|  | Social credibility (insurance coverage) | Japan | 518 | 3.63 | 0.67 | 525 | 3.63 | 0.66 |
|  |  | Ireland | 150 | 3.15 | 0.95 | 163 | 3.14 | 0.92 |
|  |  | Finland | 161 | 3.34 | 0.83 | 170 | 3.32 | 0.81 |
|  | Durability (resistant to breaking, upgradable) | Japan | 519 | 3.74 | 0.56 | 525 | 3.74 | 0.56 |
|  |  | Ireland | 147 | 3.34 | 0.90 | 163 | 3.31 | 0.86 |
|  |  | Finland | 163 | 3.60 | 0.76 | 170 | 3.59 | 0.75 |
|  | Size | Japan | 519 | 3.52 | 0.69 | 525 | 3.53 | 0.68 |
|  |  | Ireland | 138 | 3.14 | 0.80 | 163 | 3.12 | 0.74 |
|  |  | Finland | 154 | 3.02 | 0.88 | 170 | 3.02 | 0.84 |
|  | Design (appearance, color, shape, materials) | Japan | 520 | 3.13 | 0.80 | 525 | 3.13 | 0.80 |
|  |  | Ireland | 146 | 3.03 | 0.79 | 163 | 3.03 | 0.75 |
|  |  | Finland | 164 | 2.68 | 0.89 | 170 | 2.69 | 0.87 |
|  | Popularity and reputation | Japan | 517 | 3.06 | 0.84 | 525 | 3.06 | 0.83 |
|  |  | Ireland | 148 | 2.95 | 0.84 | 163 | 2.96 | 0.80 |
|  |  | Finland | 162 | 2.69 | 0.87 | 170 | 2.70 | 0.86 |
|  | Opinions of people close to you | Japan | 519 | 2.98 | 0.86 | 525 | 2.98 | 0.85 |
|  |  | Ireland | 145 | 2.93 | 0.90 | 163 | 2.94 | 0.85 |
|  |  | Finland | 154 | 2.72 | 0.93 | 170 | 2.75 | 0.89 |
|  | Information and instructions (special features, users’ manual) | Japan | 519 | 3.47 | 0.68 | 525 | 3.47 | 0.68 |
|  |  | Ireland | 146 | 3.36 | 0.89 | 163 | 3.32 | 0.84 |
|  |  | Finland | 159 | 3.49 | 0.86 | 170 | 3.48 | 0.84 |
|  | Law and regulation (responsibility in case of accidents) | Japan | 520 | 3.71 | 0.57 | 525 | 3.71 | 0.57 |
|  |  | Ireland | 145 | 3.42 | 0.89 | 163 | 3.37 | 0.85 |
|  |  | Finland | 162 | 3.55 | 0.83 | 170 | 3.56 | 0.81 |
|  | Capacity to increase mental and physical wellbeing and comfort | Japan | 518 | 3.34 | 0.75 | 525 | 3.34 | 0.75 |
|  |  | Ireland | 146 | 3.45 | 0.82 | 163 | 3.40 | 0.79 |
|  |  | Finland | 163 | 3.34 | 0.93 | 170 | 3.34 | 0.91 |
|  | Guarantee of entitlement to receiving human care, irrespective of the use of home-care robots | Japan | 520 | 3.69 | 0.59 | 525 | 3.70 | 0.59 |
|  |  | Ireland | 147 | 3.54 | 0.83 | 163 | 3.58 | 0.80 |
|  |  | Finland | 164 | 3.74 | 0.69 | 170 | 3.75 | 0.68 |
|  |  |  |  |  |  |  |  |  |
| **Functions expected from home-care robots** | | | | | | | | |
|  | Notifying family members and support personnel when an unexpected change occurs in an older person | Japan | 517 | 3.73 | 0.57 | 525 | 3.73 | 0.57 |
|  |  | Ireland | 153 | 3.63 | 0.75 | 163 | 3.65 | 0.73 |
|  |  | Finland | 163 | 3.65 | 0.71 | 170 | 3.66 | 0.70 |
|  | Informing an older person of the things they should be doing at the scheduled time or date (e.g., take medications) | Japan | 517 | 3.67 | 0.65 | 525 | 3.68 | 0.65 |
|  |  | Ireland | 150 | 3.59 | 0.73 | 163 | 3.62 | 0.71 |
|  |  | Finland | 163 | 3.61 | 0.77 | 170 | 3.62 | 0.76 |
|  | Providing support for the movement/mobility of older people in their daily lives | Japan | 515 | 3.48 | 0.73 | 525 | 3.47 | 0.73 |
|  |  | Ireland | 149 | 3.54 | 0.76 | 163 | 3.58 | 0.74 |
|  |  | Finland | 161 | 3.33 | 0.89 | 170 | 3.32 | 0.87 |
|  | Accompanying an older person on outings, from the time they leave home until they return | Japan | 514 | 2.94 | 0.95 | 525 | 2.94 | 0.94 |
|  |  | Ireland | 150 | 3.08 | 0.92 | 163 | 3.07 | 0.88 |
|  |  | Finland | 160 | 3.04 | 1.00 | 170 | 3.04 | 0.97 |
|  | Observing and recording signs that affect the mental and physical condition of an older person | Japan | 516 | 3.48 | 0.76 | 525 | 3.47 | 0.75 |
|  |  | Ireland | 149 | 3.26 | 0.83 | 163 | 3.24 | 0.80 |
|  |  | Finland | 163 | 3.32 | 0.87 | 170 | 3.31 | 0.85 |
|  | Observing and recording dementia-related symptoms | Japan | 515 | 3.36 | 0.81 | 525 | 3.35 | 0.80 |
|  |  | Ireland | 149 | 3.40 | 0.81 | 163 | 3.41 | 0.79 |
|  |  | Finland | 162 | 3.45 | 0.75 | 170 | 3.44 | 0.74 |
|  | Observing and recording the status of an older person at night | Japan | 515 | 3.49 | 0.76 | 525 | 3.48 | 0.76 |
|  |  | Ireland | 149 | 3.44 | 0.81 | 163 | 3.45 | 0.79 |
|  |  | Finland | 163 | 3.34 | 0.86 | 170 | 3.34 | 0.84 |
|  | Confirming that an older person has taken his/her medication as prescribed by a physician | Japan | 512 | 3.53 | 0.72 | 525 | 3.53 | 0.72 |
|  |  | Ireland | 150 | 3.57 | 0.75 | 163 | 3.61 | 0.73 |
|  |  | Finland | 163 | 3.63 | 0.70 | 170 | 3.64 | 0.69 |
|  | Conversing with an older person about his/her concerns and providing companionship | Japan | 516 | 3.27 | 0.80 | 525 | 3.27 | 0.79 |
|  |  | Ireland | 150 | 2.97 | 1.01 | 163 | 2.97 | 0.97 |
|  |  | Finland | 162 | 2.87 | 1.03 | 170 | 2.88 | 1.01 |
|  | Informing family members and support personnel of an older person’s concerns | Japan | 514 | 3.19 | 0.86 | 525 | 3.19 | 0.85 |
|  |  | Ireland | 148 | 3.11 | 0.98 | 163 | 3.10 | 0.94 |
|  |  | Finland | 163 | 3.22 | 0.88 | 170 | 3.21 | 0.87 |
|  | Taking care of pets (e.g., feeding and cleaning litter boxes) | Japan | 515 | 2.63 | 1.10 | 525 | 2.63 | 1.09 |
|  |  | Ireland | 146 | 2.92 | 1.01 | 163 | 2.93 | 0.96 |
|  |  | Finland | 160 | 2.44 | 1.01 | 170 | 2.41 | 0.99 |
|  | Notifying the older person as well as family members and support personnel about food that has expired | Japan | 517 | 2.97 | 0.96 | 525 | 2.97 | 0.95 |
|  |  | Ireland | 149 | 3.35 | 0.92 | 163 | 3.34 | 0.88 |
|  |  | Finland | 160 | 2.91 | 0.93 | 170 | 2.92 | 0.90 |
|  | Detecting obstacles on the floor to prevent falls | Japan | 516 | 3.43 | 0.76 | 525 | 3.43 | 0.76 |
|  |  | Ireland | 149 | 3.70 | 0.66 | 163 | 3.72 | 0.63 |
|  |  | Finland | 163 | 3.49 | 0.76 | 170 | 3.49 | 0.75 |
|  | Notifying family members or support personnel of a home intrusion by a suspicious individual(s) | Japan | 514 | 3.62 | 0.71 | 525 | 3.63 | 0.70 |
|  |  | Ireland | 149 | 3.79 | 0.62 | 163 | 3.81 | 0.59 |
|  |  | Finland | 162 | 3.80 | 0.58 | 170 | 3.81 | 0.57 |
|  | Implementing cognitive function training (brain training) | Japan | 511 | 3.27 | 0.85 | 525 | 3.26 | 0.84 |
|  |  | Ireland | 147 | 3.34 | 0.84 | 163 | 3.31 | 0.80 |
|  |  | Finland | 163 | 3.36 | 0.85 | 170 | 3.34 | 0.84 |
|  |  |  |  |  |  |  |  |  |
| **Ethically acceptable uses** | | | | | | | | |
|  | Home-care robots should be allowed to take photos or record videos that can identify the user, with his/her permission. | Japan | 519 | 2.66 | 1.02 | 525 | 2.67 | 1.01 |
|  |  | Ireland | 152 | 2.34 | 1.09 | 163 | 2.31 | 1.05 |
|  |  | Finland | 165 | 2.38 | 1.05 | 170 | 2.38 | 1.03 |
|  | Home-care robots should be allowed to take photos or record videos as long as the individual cannot be identified (by blurring so that images show only silhouettes or by converting to text). | Japan | 518 | 2.65 | 0.96 | 525 | 2.65 | 0.95 |
|  |  | Ireland | 153 | 2.41 | 1.03 | 163 | 2.39 | 1.00 |
|  |  | Finland | 164 | 2.57 | 0.98 | 170 | 2.58 | 0.97 |
|  | Healthcare professionals should be allowed to use photos and videos recorded by home-care robots for clinical care and monitoring. | Japan | 519 | 2.76 | 0.93 | 525 | 2.77 | 0.93 |
|  |  | Ireland | 155 | 2.56 | 1.03 | 163 | 2.58 | 1.01 |
|  |  | Finland | 162 | 2.78 | 0.98 | 170 | 2.77 | 0.97 |
|  | Health care professionals should be allowed to receive information on vital signs obtained by a home-care robot (e.g., blood pressure, body temperature, respiration, pulse). | Japan | 520 | 3.46 | 0.73 | 525 | 3.46 | 0.73 |
|  |  | Ireland | 155 | 3.25 | 0.89 | 163 | 3.23 | 0.87 |
|  |  | Finland | 163 | 3.44 | 0.88 | 170 | 3.43 | 0.87 |
|  | Healthcare professionals should be allowed to use verbal information obtained by a home-care robot from the user. | Japan | 520 | 3.04 | 0.91 | 525 | 3.04 | 0.91 |
|  |  | Ireland | 153 | 2.91 | 0.93 | 163 | 2.91 | 0.91 |
|  |  | Finland | 164 | 2.79 | 1.04 | 170 | 2.79 | 1.03 |
|  | Healthcare professionals should be allowed to use information about the user's location obtained by a home-care robot. | Japan | 516 | 3.08 | 0.89 | 525 | 3.08 | 0.88 |
|  |  | Ireland | 153 | 3.01 | 0.97 | 163 | 3.01 | 0.94 |
|  |  | Finland | 160 | 3.13 | 1.01 | 170 | 3.12 | 0.98 |
|  | Healthcare professionals should be allowed to use secondary information (e.g., blurred images and analyzed data) collected by a home-care robot. | Japan | 520 | 2.78 | 0.92 | 525 | 2.79 | 0.92 |
|  |  | Ireland | 151 | 2.63 | 1.01 | 163 | 2.66 | 0.98 |
|  |  | Finland | 161 | 2.92 | 1.03 | 170 | 2.92 | 1.00 |
|  | Researchers should be allowed to use secondary information (e.g., blurred images and analyzed data) collected by a home-care robot. | Japan | 520 | 2.79 | 0.92 | 525 | 2.79 | 0.91 |
|  |  | Ireland | 147 | 2.54 | 1.04 | 163 | 2.58 | 1.00 |
|  |  | Finland | 162 | 2.74 | 1.05 | 170 | 2.75 | 1.03 |
|  | I want to help other people and society by participating in the research and development of home-care robots. | Japan | 519 | 2.91 | 0.94 | 525 | 2.91 | 0.93 |
|  |  | Ireland | 150 | 3.17 | 0.97 | 163 | 3.16 | 0.93 |
|  |  | Finland | 163 | 2.86 | 1.07 | 170 | 2.86 | 1.04 |
|  | I am open to using a home-care robot even during the research and development stage if it would benefit me. | Japan | 520 | 2.84 | 0.97 | 525 | 2.84 | 0.96 |
|  |  | Ireland | 152 | 3.05 | 1.06 | 163 | 3.04 | 1.03 |
|  |  | Finland | 163 | 2.61 | 1.13 | 170 | 2.62 | 1.11 |

SD: standard deviation

**Appendix Table 2. Items associated with willingness to use home-care robots: Bivariate analysis**

|  |  | Japan n=525 | | | | | | Ireland n=163 | | | | | | Finland n=170 | | | | | |
| --- | --- | --- | --- | --- | --- | --- | --- | --- | --- | --- | --- | --- | --- | --- | --- | --- | --- | --- | --- |
|  |  | I would like to use one. | | I would not want to use one. | |  | | I would like to use one. | | I would not want to use one. | |  | | I would like to use one. | | I would not want to use one. | |  | |
|  |  | n=377 | | n=148 | |  | | n=113 | | n=50 | |  | | n=90 | | n=80 | |  | |
|  |  | n | % | n | % | p-value | Cronbach’s alpha | n | % | n | % | p-value | Cronbach’s alpha | n | % | n | % | p-value | Cronbach’s alpha |
| **Familiarity with robots ^a^** | |  |  |  |  |  | 0.67 |  |  |  |  |  | 0.61 |  |  |  |  |  | 0.66 |
|  | I have seen news about robots. | 366 | 97.1 | 127 | 85.8 | <.001 |  | 86 | 76.1 | 33 | 66.0 | 0.18 |  | 71 | 78.9 | 52 | 65.0 | 0.043 |  |
|  | I am interested in robot-related news. | 353 | 93.6 | 96 | 64.9 | <.001 |  | 106 | 93.8 | 23 | 46.0 | <.001 |  | 75 | 83.3 | 34 | 42.5 | <.001 |  |
|  | I have seen a robot that was developed for caregiving. | 144 | 38.2 | 29 | 19.6 | <.001 |  | 38 | 33.6 | 15 | 30.0 | 0.65 |  | 34 | 37.8 | 18 | 22.5 | 0.031 |  |
|  | I have used a robot that was developed for caregiving. | 41 | 10.9 | 6 | 4.1 | 0.01 |  | 7 | 6.2 | 2 | 4.0 | 0.72 |  | 5 | 5.6 | 3 | 3.8 | 0.724 |  |
|  | I have seen an animation/manga in which a robot appeared. | 254 | 67.4 | 66 | 44.6 | <.001 |  | 48 | 42.5 | 19 | 38.0 | 0.59 |  | 47 | 52.2 | 28 | 35.0 | 0.024 |  |
|  | I have a positive impression of robots. | 342 | 90.7 | 98 | 66.2 | <.001 |  | 98 | 86.7 | 24 | 48.0 | <.001 |  | 66 | 73.3 | 27 | 33.8 | <.001 |  |
|  | I have used a robot such as an animal-like robot, a humanoid robot or a cleaning robot. | 109 | 28.9 | 21 | 14.2 | <.001 |  | 11 | 9.7 | 3 | 6.0 | 0.43 |  | 15 | 16.7 | 9 | 11.3 | 0.311 |  |
|  |  |  |  |  |  |  |  |  |  |  |  |  |  |  |  |  |  |  |  |
| **Important points about home-care robots ^a^** | | |  |  |  |  | 0.91 |  |  |  |  |  | 0.95 |  |  |  |  |  | 0.94 |
|  | Convenience (ease of use) | 368 | 97.6 | 122 | 82.4 | <.001 |  | 106 | 93.8 | 33 | 66.0 | <.001 |  | 87 | 96.7 | 61 | 76.3 | <.001 |  |
|  | Entertainment value (fun to use) | 302 | 80.1 | 99 | 66.9 | <.001 |  | 90 | 79.6 | 27 | 54.0 | <.001 |  | 68 | 75.6 | 43 | 53.8 | <.001 |  |
|  | Economic efficiency (low running cost) | 372 | 98.7 | 135 | 91.2 | <.001 |  | 103 | 91.2 | 29 | 58.0 | <.001 |  | 88 | 97.8 | 62 | 77.5 | <.001 |  |
|  | Safety (warranty, after-service) | 376 | 99.7 | 138 | 93.2 | <.001 |  | 107 | 94.7 | 34 | 68.0 | <.001 |  | 89 | 98.9 | 63 | 78.8 | <.001 |  |
|  | High performance and capability | 374 | 99.2 | 136 | 91.9 | <.001 |  | 105 | 92.9 | 33 | 66.0 | <.001 |  | 83 | 92.2 | 59 | 73.8 | <.001 |  |
|  | Confidentiality (privacy protection) | 359 | 95.2 | 127 | 85.8 | <.001 |  | 105 | 92.9 | 33 | 66.0 | <.001 |  | 85 | 94.4 | 64 | 80.0 | <.001 |  |
|  | Social credibility (HSE-accredited) | 361 | 95.8 | 128 | 86.5 | <.001 |  | 102 | 90.3 | 29 | 58.0 | <.001 |  | 88 | 97.8 | 61 | 76.3 | <.001 |  |
|  | Durability (resistant to breaking, upgradable) | 374 | 99.2 | 135 | 91.2 | <.001 |  | 107 | 94.7 | 34 | 68.0 | <.001 |  | 88 | 97.8 | 67 | 83.8 | <.001 |  |
|  | Size | 355 | 94.2 | 133 | 89.9 | 0.08 |  | 108 | 95.6 | 33 | 66.0 | <.001 |  | 81 | 90.0 | 55 | 68.8 | <.001 |  |
|  | Design (appearance, color, shape, materials) | 308 | 81.7 | 110 | 74.3 | 0.06 |  | 102 | 90.3 | 36 | 72.0 | <.001 |  | 70 | 77.8 | 39 | 48.8 | <.001 |  |
|  | Popularity and reputation | 288 | 76.4 | 115 | 77.7 | 0.75 |  | 99 | 87.6 | 30 | 60.0 | <.001 |  | 67 | 74.4 | 42 | 52.5 | <.001 |  |
|  | Opinions of people close to you | 279 | 74.0 | 109 | 73.6 | 0.93 |  | 94 | 83.2 | 29 | 58.0 | <.001 |  | 61 | 67.8 | 49 | 61.3 | 0.37 |  |
|  | Information and instructions (special features, users’ manual) | 358 | 95.0 | 128 | 86.5 | <.001 |  | 106 | 93.8 | 35 | 70.0 | <.001 |  | 85 | 94.4 | 65 | 81.3 | 0.01 |  |
|  | Law and regulation (responsibility in case of accidents) | 371 | 98.4 | 133 | 89.9 | <.001 |  | 105 | 92.9 | 37 | 74.0 | <.001 |  | 87 | 96.7 | 66 | 82.5 | <.001 |  |
|  | Capacity to increase mental and physical wellbeing and comfort | 340 | 90.2 | 123 | 83.1 | 0.02 |  | 110 | 97.3 | 38 | 76.0 | <.001 |  | 89 | 98.9 | 56 | 70.0 | <.001 |  |
|  | Guarantee of entitlement to receiving human care, irrespective of the use of home-care robots | 359 | 95.2 | 140 | 94.6 | 0.76 |  | 108 | 95.6 | 39 | 78.0 | <.001 |  | 90 | 100.0 | 69 | 86.3 | <.001 |  |
|  |  |  |  |  |  |  |  |  |  |  |  |  |  |  |  |  |  |  |  |
| **Functions expected from home-care robots ^a^** | | |  |  |  |  | 0.92 |  |  |  |  |  | 0.95 |  |  |  |  |  | 0.94 |
|  | Notifying family members and support personnel when an unexpected change occurs in an older person | 371 | 98.4 | 132 | 89.2 | <.001 |  | 111 | 98.2 | 41 | 82.0 | <.001 |  | 90 | 100.0 | 70 | 87.5 | <.001 |  |
|  | Informing an older person of the things they should be doing at the scheduled time or date (e.g., take medications) | 363 | 96.3 | 129 | 87.2 | <.001 |  | 111 | 98.2 | 42 | 84.0 | <.001 |  | 90 | 100.0 | 65 | 81.3 | <.001 |  |
|  | Providing support for the movement/mobility that older people in their daily lives | 357 | 94.7 | 118 | 79.7 | <.001 |  | 112 | 99.1 | 41 | 82.0 | <.001 |  | 87 | 96.7 | 58 | 72.5 | <.001 |  |
|  | Accompanying an older person on outings, from the time they leave home until the time they return | 263 | 69.8 | 81 | 54.7 | <.001 |  | 99 | 87.6 | 31 | 62.0 | <.001 |  | 75 | 83.3 | 51 | 63.8 | <.001 |  |
|  | Observing and recording matters that affect the mental and physical condition of an older person | 356 | 94.4 | 116 | 78.4 | <.001 |  | 104 | 92.0 | 36 | 72.0 | <.001 |  | 84 | 93.3 | 63 | 78.8 | 0.01 |  |
|  | Observing and recording dementia-related symptoms | 344 | 91.2 | 109 | 73.6 | <.001 |  | 107 | 94.7 | 39 | 78.0 | <.001 |  | 88 | 97.8 | 67 | 83.8 | <.001 |  |
|  | Observing and recording the status of an older person at night | 354 | 93.9 | 118 | 79.7 | <.001 |  | 109 | 96.5 | 38 | 76.0 | <.001 |  | 87 | 96.7 | 60 | 75.0 | <.001 |  |
|  | Confirming that an older person has taken his/her medication as prescribed by a physician | 360 | 95.5 | 120 | 81.1 | <.001 |  | 111 | 98.2 | 40 | 80.0 | <.001 |  | 90 | 100.0 | 69 | 86.3 | <.001 |  |
|  | Conversing with an older person about his/her concerns and providing companionship | 333 | 88.3 | 107 | 72.3 | <.001 |  | 97 | 85.8 | 27 | 54.0 | <.001 |  | 67 | 74.4 | 49 | 61.3 | 0.07 |  |
|  | Informing family members and support personnel of an older person’s concerns | 315 | 83.6 | 101 | 68.2 | <.001 |  | 100 | 88.5 | 31 | 62.0 | <.001 |  | 80 | 88.9 | 61 | 76.3 | 0.03 |  |
|  | Taking care of pets (e.g., feeding and cleaning litter boxes) | 222 | 58.9 | 67 | 45.3 | 0.01 |  | 91 | 80.5 | 29 | 58.0 | <.001 |  | 40 | 44.4 | 36 | 45.0 | 0.94 |  |
|  | Notifying the older person as well as family members and support personnel about food that has expired | 285 | 75.6 | 82 | 55.4 | <.001 |  | 103 | 91.2 | 37 | 74.0 | <.001 |  | 71 | 78.9 | 55 | 68.8 | 0.13 |  |
|  | Detecting obstacles on the floor to prevent falls | 350 | 92.8 | 117 | 79.1 | <.001 |  | 112 | 99.1 | 43 | 86.0 | <.001 |  | 88 | 97.8 | 68 | 85.0 | <.001 |  |
|  | Notifying family members or support personnel of a home intrusion by a suspicious individual(s) | 358 | 95.0 | 127 | 85.8 | <.001 |  | 112 | 99.1 | 43 | 86.0 | <.001 |  | 90 | 100.0 | 72 | 90.0 | <.001 |  |
|  | Implementing cognitive function training (brain training) | 317 | 84.1 | 112 | 75.7 | 0.025 |  | 106 | 93.8 | 38 | 76.0 | <.001 |  | 87 | 96.7 | 61 | 76.3 | <.001 |  |
|  |  |  |  |  |  |  |  |  |  |  |  |  |  |  |  |  |  |  |  |
|  |  | m | SD | m | SD | p-value |  | m | SD | m | SD | p-value |  | m | SD | m | SD | p-value |  |
| **Ethically acceptable uses ^b^** | |  |  |  |  |  | 0.87 |  |  |  |  |  | 0.89 |  |  |  |  |  | 0.90 |
|  | Acquisition of personal information | 8.37 | 2.3 | 7.31 | 2.3 | <.001 |  | 7.71 | 2.5 | 6.4 | 2.8 | <.001 |  | 8.49 | 1.9 | 6.86 | 2.6 | <.001 |  |
|  | Use of personal information for medical and long-term care | 9.81 | 2.1 | 8.99 | 2.3 | <.001 |  | 9.71 | 2.0 | 7.91 | 2.5 | <.001 |  | 10.18 | 1.8 | 8.4 | 2.8 | <.001 |  |
|  | Secondary use of personal information | 5.72 | 1.7 | 5.2 | 1.7 | <.001 |  | 5.52 | 1.7 | 4.46 | 2.0 | <.001 |  | 6.32 | 1.5 | 4.92 | 2.1 | <.001 |  |
|  | Willingness to participate in research and development | 6.18 | 1.5 | 4.63 | 1.9 | <.001 |  | 6.87 | 1.3 | 4.77 | 2.2 | <.001 |  | 6.46 | 1.6 | 4.36 | 1.7 | <.001 |  |

^a)^ χ² test or Fisher exact test

^b)^ t-test

m: mean; SD: standard deviation

**Appendix Table 3. Factors associated with willingness to use home-care robots in each category: Logistic regression analysis**

|  | **Independent variable** | Japan n=525 | | Ireland n=163 | | Finland n=170 | |
| --- | --- | --- | --- | --- | --- | --- | --- |
|  |  | odds ratio | 95%CI | odds ratio | 95%CI | odds ratio | 95%CI |
| **Familiarity with robots** | |  |  |  |  |  |  |
|  | I am interested in robot-related news. | 6.57 | 3.71-11.61 | 25.31 | 8.62-74.32 | 5.21 | 2.47-10.99 |
|  | I have seen a robot that was developed for caregiving. | 1.99 | 1.19-3.31 |  |  |  |  |
|  | I have seen an animation/manga in which a robot appeared. | 1.75 | 1.12-2.73 |  |  |  |  |
|  | I have a positive impression of robots. | 5.00 | 2.93-8.52 | 10.65 | 4.07-27.82 | 4.09 | 2.02-8.26 |
| **Important points about home-care robots** | |  |  |  |  |  |  |
|  | Convenience | 6.93 | 3.09-15.57 |  |  |  |  |
|  | Economic efficiency |  |  | 4.44 | 1.74-11.37 |  |  |
|  | Size |  |  | 6.24 | 1.97-19.73 |  |  |
|  | Design |  |  |  |  | 2.27 | 1.10-4.69 |
|  | Law and regulation | 4.23 | 1.49-11.99 |  |  |  |  |
|  | Capacity to increase mental and physical wellbeing and comfort |  |  |  |  | 26.72 | 3.44-207.75 |
| **Functions expected from home-care robots** | |  |  |  |  |  |  |
|  | Notifying family members and support personnel when an unexpected change occurs in an older person |  |  | 11.15 | 2.18-57.15 |  |  |
|  | Providing support for the movements/mobility that older people regularly carry out in their daily lives | 2.43 | 1.22-4.82 |  |  | 5.91 | 1.58-22.14 |
|  | Observing and recording matters that affect the mental and physical condition of an older person | 2.80 | 1.45-5.42 |  |  |  |  |
|  | Conversing with an older person about his/her concerns and providing companionship | 2.03 | 1.21-3.41 | 4.94 | 2.23-10.97 |  |  |
| **Ethically acceptable uses** | |  |  |  |  |  |  |
|  | Acquisition of personal information | 2.00 | 1.34-3.01 |  |  |  |  |
|  | Use of personal information for medical and long-term care |  |  | 3.00 | 1.33-6.76 |  |  |
|  | Willingness to participate in research and development | 3.76 | 2.51-5.64 | 6.08 | 2.74-13.51 | 7.34 | 3.69-14.57 |

CI: confidence interval
